# Vegetation phenology gradients along the west and east coasts of Greenland from 2001 to 2015

**DOI:** 10.1007/s13280-016-0866-6

**Published:** 2017-01-23

**Authors:** Mojtaba Karami, Birger Ulf Hansen, Andreas Westergaard-Nielsen, Jakob Abermann, Magnus Lund, Niels Martin Schmidt, Bo Elberling

**Affiliations:** 1 Department of Geosciences and Natural Resource Management, Center for Permafrost (CENPERM), University of Copenhagen, Øster Voldgade 10, 1350 Copenhagen K, Denmark; 2 Asiaq, Greenland Survey, Qatserisut 8, 3900 Nuuk, Greenland; 3 Department of Bioscience, Arctic Research Centre, Aarhus University, Frederiksborgvej 399, 4000 Roskilde, Denmark

**Keywords:** Arctic climate, Greenland phenology, Land surface temperature, Sea ice, Tundra vegetation

## Abstract

The objective of this paper is to characterize the spatiotemporal variations of vegetation phenology along latitudinal and altitudinal gradients in Greenland, and to examine local and regional climatic drivers. Time-series from the Moderate Resolution Imaging Spectroradiometer (MODIS) were analyzed to obtain various phenological metrics for the period 2001–2015. MODIS-derived land surface temperatures were corrected for the sampling biases caused by cloud cover. Results indicate significant differences between West and East Greenland, in terms of both observed phenology during the study period, as well as the climatic response. The date of the start of season (SOS) was significantly earlier (24 days), length of season longer (25 days), and time-integrated NDVI higher in West Greenland. The sea ice concentration during May was found to have a significant effect on the date of the SOS only in West Greenland, with the strongest linkage detected in mid-western parts of Greenland.

## Introduction

Vegetation phenology plays an important role in regulating ecosystem processes across the arctic ecosystems which have experienced the most marked climate change in the past 10–20 years (IPCC 2013). Here, the timings of onset and end of the growing season correspond to the start and end of the carbon uptake period, and as a result directly influence the carbon cycle (Goulden 1998; Barichivich et al. 2013; Westergaard-Nielsen et al. 2013). Vegetation phenology can also control water and energy exchanges with the atmosphere through various feedback mechanisms (Penuelas et al. 2009; Kasurinen et al. 2014; Zhang et al. 2014). Moreover, where the climate-induced shifts in phenology differ among various species, these changes can potentially result in dire consequences for species interactions (Forrest and Miller-Rushing 2010; Schmidt et al. 2016). Therefore, understanding the linkages between climatic variables and vegetation phenology lies at the core of making sound predictions about climate-induced changes in the Arctic.

Remotely sensed datasets of vegetation indices have been extensively used to investigate vegetation phenology and its changes in the Arctic at various scales (Beck and Goetz 2012; Bhatt et al. 2013; Karlsen et al. 2014), which have highlighted the influence of both large- and local-scale climate (Bhatt et al. 2010; Bieniek et al. 2015; Young et al. 2016).

Specifically in Greenland, studies have focused on understanding the drivers of vegetation dynamics (Tamstorf et al. 2007; Kerby and Post 2013). These have mostly investigated the relationship between phenology and various climatic indicators based on long-term plot-level observations or experiments. There have also been a number of attempts at upscaling the plot observations to landscape level (Westergaard-Nielsen et al. 2013). Such studies up to landscape scales are advantageous in the fact that due to the possibility of accessing the study sites, a variety of climatic parameters (e.g., temperatures, precipitation, soil moisture, and snow depth) can be monitored, and the landscape-level heterogeneity (e.g., effects of hydrology, microtopography, and vegetation patches) can be taken into consideration. However, it remains unclear as to what extent the developed models are scalable to all regions across Greenland or other parts of the Arctic. Furthermore, regional-level studies are needed to properly assess large-scale interactions between the climate and vegetation in Greenland, for instance sea ice dynamics which are shown to not only have feedback effects on the global climate, but also more directly on coastal areas of the Arctic (Bhatt et al. 2010; Dutrieux et al. 2012).

In this study, we quantify the spatial variability of vegetation phenology and surface temperatures along environmental gradients in Greenland. Subsequently, based on the data from the period 2001–2015, we address the question of whether the climate response of the vegetation in Greenland has been significantly different across various regions and altitudes. For this purpose, remotely sensed data from NASA’s Moderate Resolution Imaging Spectroradiometer (MODIS) are used to investigate the changes in mean surface temperatures and vegetation phenology from south to north and along the east and west coasts of Greenland. The main focus of this study is therefore on the regional scale variability of surface temperatures and phenology, and identifying the processes driving the spatial variability of phenology at these scales.

## Materials and methods

### Study area and in situ data

**Fig. 1:**
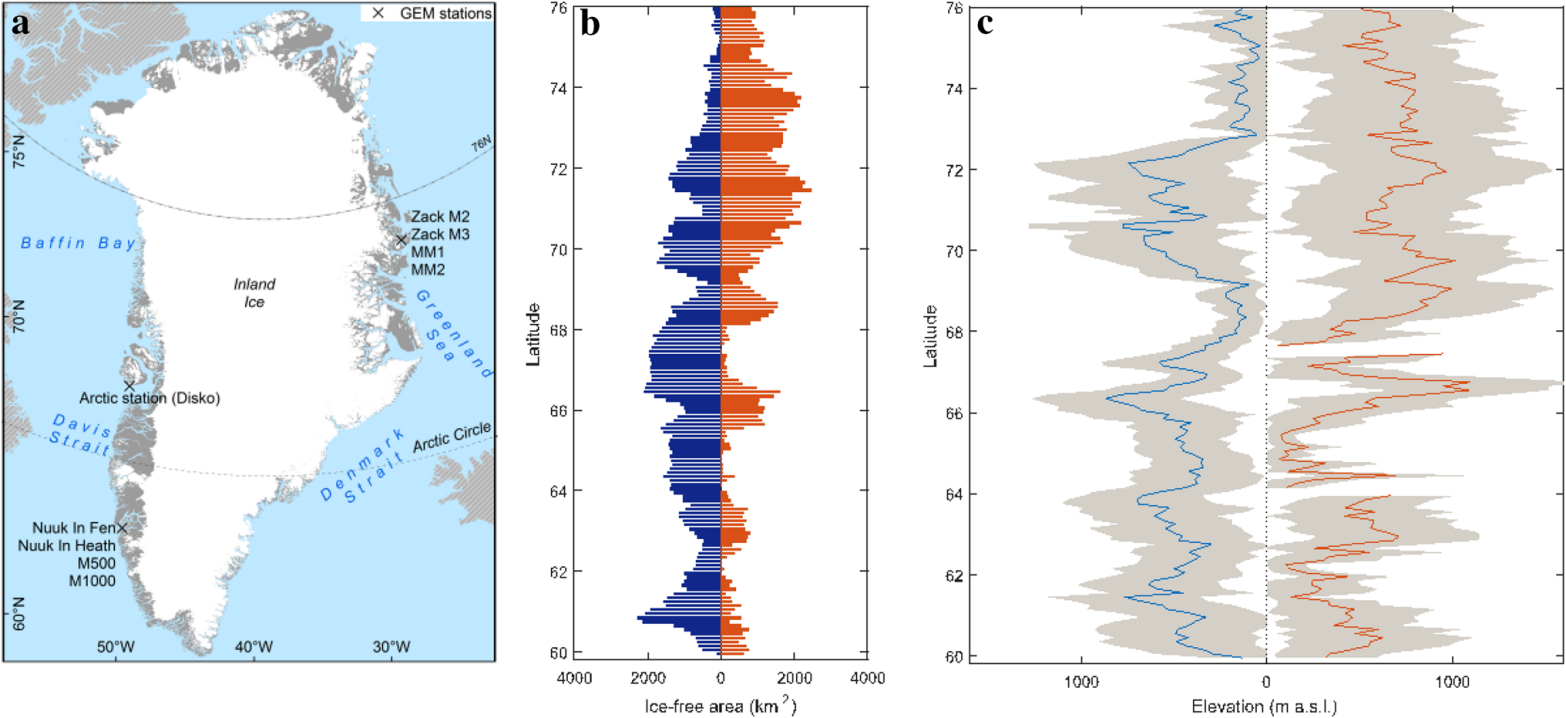
**a** Map of Greenland with the location of GEM stations. **b** Total ice-free area in East and West Greenland. The area is calculated based on sinusoidal projection. **c** Average elevation of the ice-free areas in West and East Greenland, based on digital elevation data from Howat et al. (2014). The *gray shade* represents the standard deviation

Greenland is the world’s largest island stretching over almost 24° of latitude or more than 2600 km from south to north, and 1000 km from east to west (Fig. 1a).

In East Greenland, the East Greenland Current transports cold, ice-rich, low salinity waters from the Arctic Ocean southwards along the coast. East Greenland Current is slowly mixed with branches of North Atlantic currents, gradually reducing the size of the sea ice that reaches the southern tip of Greenland (Cappelen 2012). A branch of this mixed current makes its way northwards along the west coast of Greenland, forming the West Greenland Current. In the winter, most of the Baffin Bay is covered by 3–4 m thick ice which is almost completely melted away during the summer. During the ice season, there is always more ice in the western than in the eastern parts of the Baffin Bay, due to the effect of the warm West Greenland Current. The melting of ice usually starts in April close to the west coast of Greenland, and gradually propagates northwards until most of the Baffin Bay is ice-free (Tang et al. 2004; Cappelen 2012).

Nearly 80 % of the surface of Greenland is covered by an ice sheet, leaving an area of 410 000 km^2^ around the island subjected to winter freezing and summer thawing. For the most part of our study, however, we have limited our analysis domain to the seasonally thawing areas from 59° to 76°N. This is because (i) the vegetation dynamic is only seen in these areas, (ii) north of 76°N, Visible/NIR data from the satellite are either unreliable due to sensor/illumination geometry or totally missing (e.g., h16v00 and h17v00 scenes in MODIS collection 5), and (iii) as exemplified by Østby et al. (2014), the landmask used in the processing chain of the collection 5 of MODIS land surface temperature (LST) products has remarkably large spatial errors in high northern latitudes.

The in situ data on radiation balance and surface temperatures were obtained from seven automatic Greenland Ecosystem Monitoring (GEM) meteorological stations across Greenland: Zackenberg (stations M2, M3, MM1, and MM2), Nuuk (stations I5Nf_Fen and I6Nh_Heath), and Disko Island (station AWS-2 Østerlien). In situ land surface temperatures were measured from infrared remote temperature sensors measuring skin temperature, and if not available from ground temperature sensors mounted closest to the surface (depth <5 cm). Subsequently, net shortwave radiation was rescaled to daily resolution and instantaneous LSTs corresponding to the MODIS overpass time were extracted. These data were used to calibrate and validate the correction method applied to the MODIS daily LST dataset.

### Satellite data

#### Vegetation indices and phenology

Time-series of 16-day vegetation indices composites from MODIS (MOD13A2.V005) were processed to extract vegetation phenology and greenness. The commonly used time-series analysis software TIMESAT was not used in this study, since the latest version was not able to directly incorporate the observation date layer in MOD13A2 and assumed uniform temporal steps of 16 days between NDVI data points. The errors arising from this assumption are discussed in more details in Bachoo et al. (2007).

The phenology algorithm developed for this study performs the following steps on the NDVI time-series: generation of a vegetation mask, smoothing and removal of cloud-induced noise, and extraction of phenological metrics for each year. The purpose of the vegetation mask is to limit the subsequent processing steps to the vegetated areas, and therefore to reduce the computational load. A simple thresholding approach was used for the vegetation mask based on the long-term average of the annual peak NDVI ($\overline{\text{NDVI}}_{ \hbox{max} } \ge 0.08$), standard deviation of the annual peak NDVI ($\sigma_{{\overline{\text{NDVI}}_{ \hbox{max} } }} \le 0.18$), and the availability of valid observations in the time-series (Ratio_valid_ ≥ 0.7). The effect of clouds on the NDVI time-series is usually noted as a sudden drop in NDVI followed by a return to normal levels. Thus, for the noise-removal, we used a step-by-step procedure to remove downward spikes from the time-series through comparing each value with the average of the adjacent data points.

Subsequently, the vegetation algorithm identifies the magnitude and timing of peak NDVI for each year. Both absolute and relative threshold values have previously been used to detect greenup and senescence dates (White et al. 2009; Garonna et al. 2014). NDVI values in the study area are controlled by both phenology and cover fraction. As a result, pixels with identical phenology may have substantially different NDVI values, which can limit the use of absolute thresholds. Consequently, a relative threshold level of 0.45 of the annual peak value was applied to highlight both greenup and senescence dates. Length of the growing season (LOS) was calculated as the difference between start of season (SOS) and end of season (EOS), while Time-Integrated NDVI (TINDVI) was calculated as the integration of interpolated NDVIs above the baseline for each year.

#### Surface temperatures

Time-series of daily LSTs from the MODIS sensor onboard NASA’s Terra satellite were acquired for Greenland covering the period from 2001 to 2015. The MOD11A1.V005 product was used which provides daytime and nighttime LSTs with 1 km^2^ spatial resolution and accuracy levels generally better than 1 K under clear-sky conditions (Wan 2008; Wan and Li 2008; Coll et al. 2009).

The averaging of daytime and nighttime observations in clear-sky conditions forms the basis of calculating climate indicators, e.g., summer warmth index (SWI) and mean seasonal/annual temperatures. However, the initial analysis on the availability of MODIS LST observations over Greenland indicated that sometimes due to prolonged cloudy conditions, clear-sky days with both daytime and nighttime observations may be concentrated more in the first or second half of the month, depending on the location and time of the year.

The abovementioned sampling bias can affect the accuracy of the derived climatic parameters in two ways. First, the skewed distribution of observations over the course of the month may impede the proper capturing of actual climatic conditions, particularly during the start and end of growing season, when surface temperatures are changing rapidly. Secondly, relying on clear-sky observations biases the climatic indicators more towards the cloud-free conditions. The first effect can be reduced—yet not completely eliminated—through temporal compositing. The second effect, however, persists unless a correction method is applied. The biases induced by clouds are also reported by Westermann et al. (2012) and Østby et al. (2014). The availability of MOD11A1 observations during 2001–2015 for one of the 8 MODIS tiles covering Greenland (i.e., h15v02) is shown in Table 1. The ideal levels for availability and skewness are 100 and 0. In the case of averaging daytime and nighttime observations, daily surface temperatures were available for less than 35 per cent of the days in all months of the year, and with an average absolute monthly skewness of 0.051 (Table 1).

**Table 1:** Monthly average percentage of days with good-quality observations and the skewness of the observation days for scene h15v02 of MODIS (2001–2015) before and after bias-correction

	J	F	M	A	M	J	J	A	S	O	N	D
% Days with valid daily LST												
Before	30.83	27.49	29.99	36.70	29.65	26.26	30.22	25.33	24.81	29.87	28.55	26.82
After	64.36	68.69	69.59	74.35	68.35	60.32	63.83	60.00	61.60	66.34	64.53	56.50
Ratio	2.09	2.50	2.32	2.02	2.30	2.30	2.11	2.37	2.48	2.22	2.26	2.10
Skewness of obs. days												
Before	0.14	−0.10	−0.05	0.06	0.04	0.04	0.06	0.01	0.02	−0.02	0.04	−0.05
After	0.05	−0.09	0.01	0.01	0.02	0.03	0.02	0.02	−0.01	−0.01	0.02	−0.04
Ratio	0.38	0.97	0.29	0.26	0.43	0.84	0.28	1.48	0.88	0.55	0.644	0.86

In order to minimize the abovementioned biases, a technique was developed to estimate the daily average surface temperature based on a linear model with two inputs: (i) modeled daily net shortwave radiation at the surface, and (ii) at least one daily LST observation from MODIS which can be either daytime or nighttime. As shown in Table 1, reducing the requirements to one daily observation (instead of two) can increase the availability of temperature readings by a factor of 2.25 while at the same time reducing the skewness of the distribution of observations by a factor of 0.63. More importantly, these improvements are almost solely due to the introduction of days with cloudy conditions in the calculations.

For model calibration, in situ data on incoming and outgoing shortwave radiation as well as LSTs were obtained from a number of GEM stations across Greenland as described earlier. Coefficients were then derived for a linear fit estimating the in situ daily average surface temperatures from in situ total net daily shortwave radiation, and instantaneous in situ surface temperatures corresponding to the Terra satellite overpass time. Three sub-models were developed in order to deal with the situations where only daytime, only nighttime, and both daytime and nighttime observations were available. For the application of the model to the satellite LST dataset, daily net shortwave radiation with 1 km resolution was calculated by combining the daily incoming shortwave radiation on the surface from ERA-interim (Dee et al. 2011) and surface albedo from MODIS albedo product (MCD43B3). Subsequently, monthly LST averages and SWI were also calculated based on the daily averages.

#### Sea ice concentration

Data on monthly sea ice concentration were extracted from NSIDC Sea Ice Index dataset. The original dataset provides sea ice concentration estimates with a resolution of 25 × 25 km based on data from passive microwave sensors. However, since the aim was to assess the regional effects of sea ice, data were resampled into a grid with a resolution of 125 × 150 km. This has resulted in 15 annual sets of 156 pixels in Baffin Bay, Davis Strait, and areas generally associated with the West Greenland, and 182 pixels in Greenland Sea, Denmark Strait, and the rest of the areas relating to Greenland’s east coast.

### Analysis

#### Relationships between phenology and surface temperatures

At the landscape scale, heterogeneity in terms of microtopography, nutrient supply, and availability of meltwater can alter the effects of LST on vegetation cover. Therefore, the effects of LST on phenology were investigated in two ways: (i) directly correlating phenology metrics to mean LST considering each 1 × 1 km pixel as an observation (herein Pixel-Based approach), and (ii) correlating regionally averaged phenology metrics to regionally averaged surface temperatures, considering bands of 0.1° latitude along each of the coasts as individual observations (regionally averaged approach). Pixel-based correlations for each region can indicate to what extent other factors than LST are influencing the phenology in that region. However, in the regional averaging approach, by using averaging and diluting the effects of measurement noise and landscape level heterogeneity, we allow the large-scale variability of LST–phenology relationship along the latitudinal gradients to emerge.

#### Phenological differences between regions

In order to answer the question of whether there are significant differences between regions in terms of phenology, the statistical significance of the differences in terms of the timing of SOS and EOS, time-integrated NDVI (TINDVI), and SWI between East and West Greenland were tested. For this purpose, regionally averaged data for analogous areas on east and west coasts along the latitudes were compared using one-way ANOVA (significant at *p* < 0.05).

#### Topographic standardization

Figure 1b, c illustrates the total ice-free area and average elevation along the latitudinal gradients on east and west coasts of Greenland, respectively. Moving from south to north, besides the average elevation, the histogram of elevation also varies from region to region—which is not shown in the figure. Given the remarkable topographic variability along each coast and also between analogous areas on the two coasts, it is expected that the observed phenological differences—or lack thereof—are partially linked to the elevation. A procedure to remove the effects of topographic difference is therefore needed in order to ascertain whether the topography has enhanced or diminished the phenological differences between the regions in Greenland. This was achieved through a weighted averaging scheme for the regionally averaged dataset, whereby pixels are first averaged within elevational classes of 100 m, and the results are then averaged giving equal weights to all elevational classes. This is akin to standardizing the elevational histogram of the regionally averaged data points by transforming all into uniform distributions.

#### Changes along altitudinal gradients

The variability of the SWI and phenology metrics (i.e., dates of start and end season, length of season, peak annual NDVI, and time-integrated NDVI) along the altitudinal gradient was analyzed using linear regression. For each latitudinal band of 1° wide on the west and east coasts of Greenland, these metrics were regressed on elevation as the independent variable. The linear slopes with no significance are subsequently removed (*p* > 0.01).

#### Sensitivity of start of season to sea ice concentrations

The effect of sea ice variation on the inter-annual variability of phenology along the two coasts of Greenland was analyzed using linear regression. The aim was to clarify how sensitive different regions are to the variations in sea ice concentration. Regionally averaged data on SOS with 0.1° latitudinal intervals for East (*n* = 236) and West Greenland (*n* = 236) were used as the response parameter. Nonetheless, the absolute range of the inter-annual fluctuations of the sea ice concentration and regionally averaged SOS can both vary spatially, thereby making it difficult to directly compare the regression-derived sensitivities among different regions. For this reason, the temporal variations of the sea ice concentrations and regionally averaged SOS were all converted to Z-scores. Linear regressions between each regionally averaged SOS data point associated with West Greenland and all SIC data points in a radius of 1° south and 4° latitude to its north were evaluated. The same procedure was then performed for the regionally averaged SOS and SIC datasets associated with East Greenland. Subsequently, non-significant relationships were removed (*p* > 0.05).

## Results

### Intra-annual dynamics of surface temperatures and phenology

**Fig. 2:**
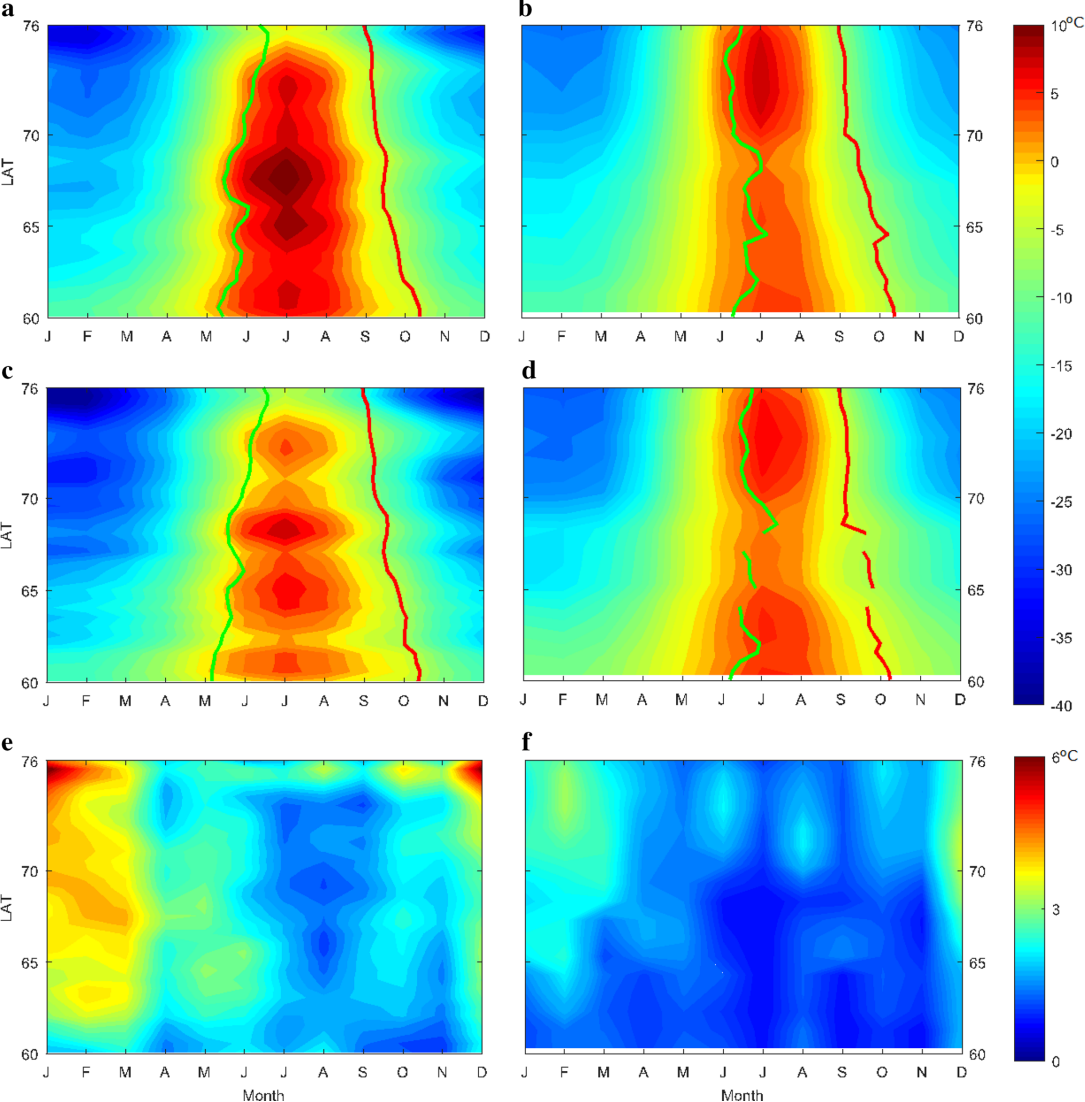
**a**, **b** Annual variations of surface temperatures in West (**a**) and East (**b**) Greenland. The average start of season date is shown as *green lines*, and the average end of season as *red lines*. **c**, **d** Annual variations of surface temperatures after topographic standardization in West (**c**) and East Greenland (**d**). **e**, **f** Temporal standard deviation of land surface temperatures in West (**e**) and East Greenland (**f**)

Figure 2 shows the latitude-time cross section for the 15-year average LSTs in East and West Greenland, as well as the temporal standard deviation of LSTs. The corresponding regionally averaged SOS and EOS dates are shown in the figure as lines. In East Greenland, the length of the thermal growing season, corresponding to the shades of red, shows a continuous latitudinal decrease from south to north. In West Greenland, the latitudinal changes of the length of the thermal growing season have some irregularities, which are presumably linked to the large variations in the average distance to the sea. Nonetheless, SOS and EOS both follow the latitudinal pattern of the thermal growing season on the two coasts, but this agreement is less visible in the case of EOS. This must be linked to the dominating effect of light availability on EOS, which has a latitudinal gradient.

Mean values represent a snapshot of the spatial variability. However, both SOS and EOS reveal substantial inter-annual variations. In West Greenland, SOS shows large inter-annual variations which are gradually reduced from south to north until around 70°N, after which the inter-annual variations remain at a constant level. This suggests that two different mechanism control SOS in West Greenland south and north of 70°N, and the mechanism in the south is more variable (presumably the West Greenland Current). On East Greenland, such latitudinal patterns are not seen in year-to-year variations of SOS.

EOS levels in East and West both reveal a steady latitudinal decrease, although with relatively small year-to-year fluctuations. The maximum EOS for each latitudinal band during the 15-year period is almost matching that of the analogous area (1-day difference on average). This level probably marks the upper limit on EOS due to light availability, and can be approximated with a linear model:

1 $$ {\text{EOS}}_{ \hbox{max} } = - 2. 9 9 {\text{ LAT}} + 4 9 1\quad (R^{2} = 0. 9 2;\;{\text{RMSE}} = 4. 3 7;\;{\text{DFE}} = 6 8). $$

### SWI–phenology linkage

**Fig. 3:**
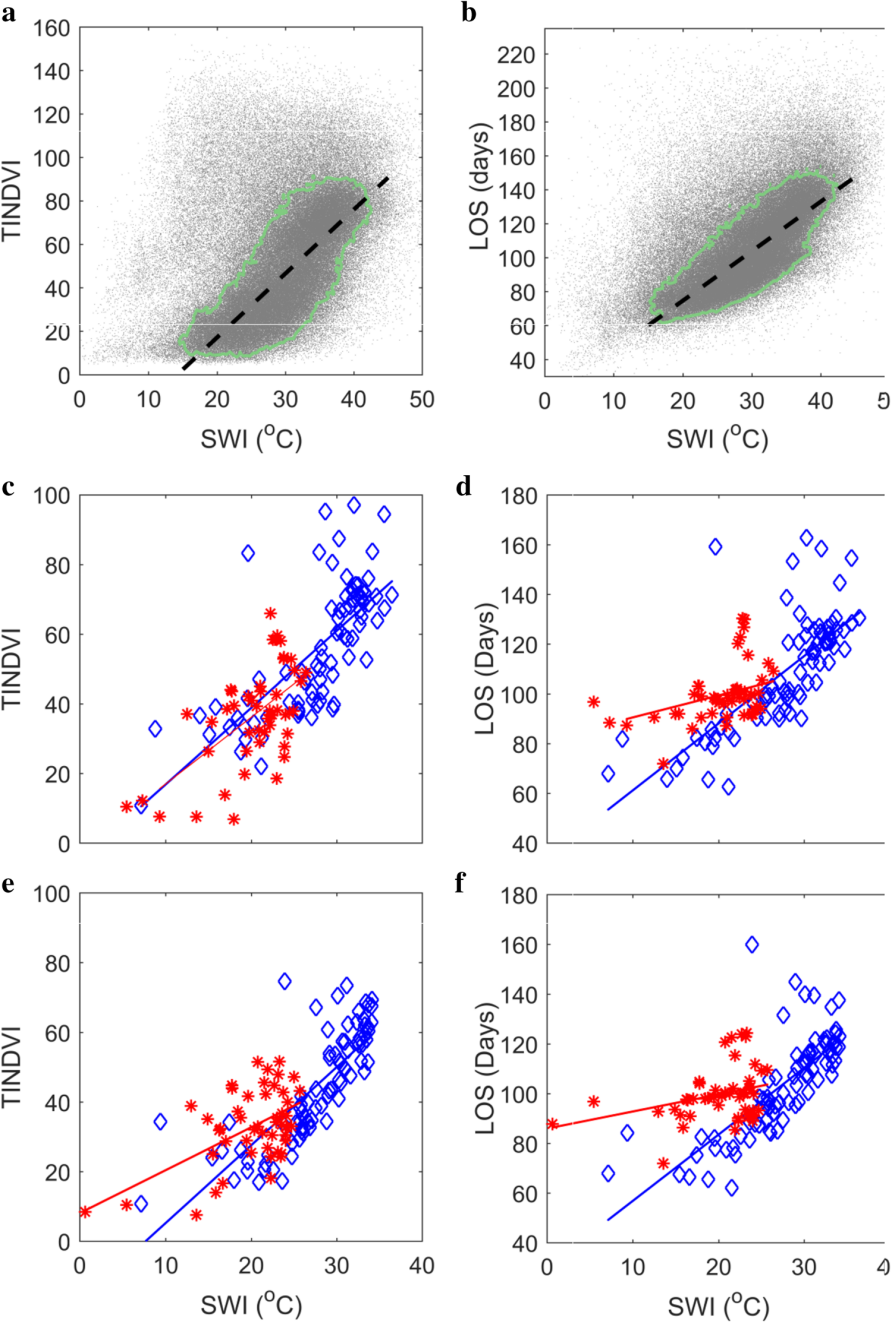
**a**, **b** Pixel-based relationship between summer warmth index, and time-integrated NDVI (**a**) and length of growing season (**b**). The *green lines* delineate the point-density threshold level based on which the outliers caused by mixed land–water/glacier pixels were excluded. **c**, **d** Relationship between summer warmth index and time-integrated NDVI (**c**) and length of growing season (**d**) based on the regionally averaged dataset. *Blue data points* and *lines* represent West Greenland and *reds* represent East Greenland. **e**, **f** Relationship between summer warmth index and time-integrated NDVI (**e**) and length of growing season (**f**) based on the regionally averaged dataset after topographic standardization. Note that the axes shown in **a**, **b** are scaled differently from those in (**c**–**f**) for visual clarity. The model results for the linear regressions are provided in Table 2

Figure 3 presents the relationships between SWI as independent, and length of growing season (LOS) and time-integrated NDVI (TINDVI) as response variables. The statistics are provided in Table 2. With regard to the pixel-based approach, the sub-pixel heterogeneities, including the presence of water bodies and glaciers might affect the reliability of the estimated parameters. Such mixed pixels can be partially removed through an NDVI-based filtering prior to the calculation of phenology metrics. Nonetheless, many outliers can still be seen on surface temperature–phenology scatterplots (Fig. 3a, b). Mapping of the pixels belonging to the low-density spaces in Fig. 3a, b suggests that the majority of these outliers correspond to the pixels located on the coastlines or margins of fjords and glaciers, and are therefore highly likely to be mixed. These pixels were considered unreliable and were excluded from the analysis.

**Table 2:** Linear regression statistics for SWI–TINDVI (bold) and SWI–LOS (regular) relationships

PB model	*R* ^2^	RMSE	slope
West Greenland RA	**0.52**	**12.82**	**2.20**
	0.53	15.61	2.7
East Greenland RA	**0.43**	**11.39**	**1.92**
	0.15	10.34	0.89
West Greenland RA-ST	**0.61**	**10.02**	**2.24**
	0.56	13.31	2.68
East Greenland RA-ST	**0.32**	**9.25**	**1.24**
	0.09	10.29	0.68
Greenland PB	**0.64**	**12.50**	**2.94**
	0.68	11.23	2.92
Iceland PB	**0.72**	**15.59**	**5.24**
	0.83	16.26	6.66

*RA* regionally averaged, *ST* standardized topography, *PB* pixel-based

In the case of the regional averaging approach, however, the deviations from the model are more linked to the differences between various regions in terms of the co-influencing factors other than temperature (e.g., water availability), which can affect the SWI–phenology relationship.

### Timing of phenology on the two coasts

**Fig. 4:**
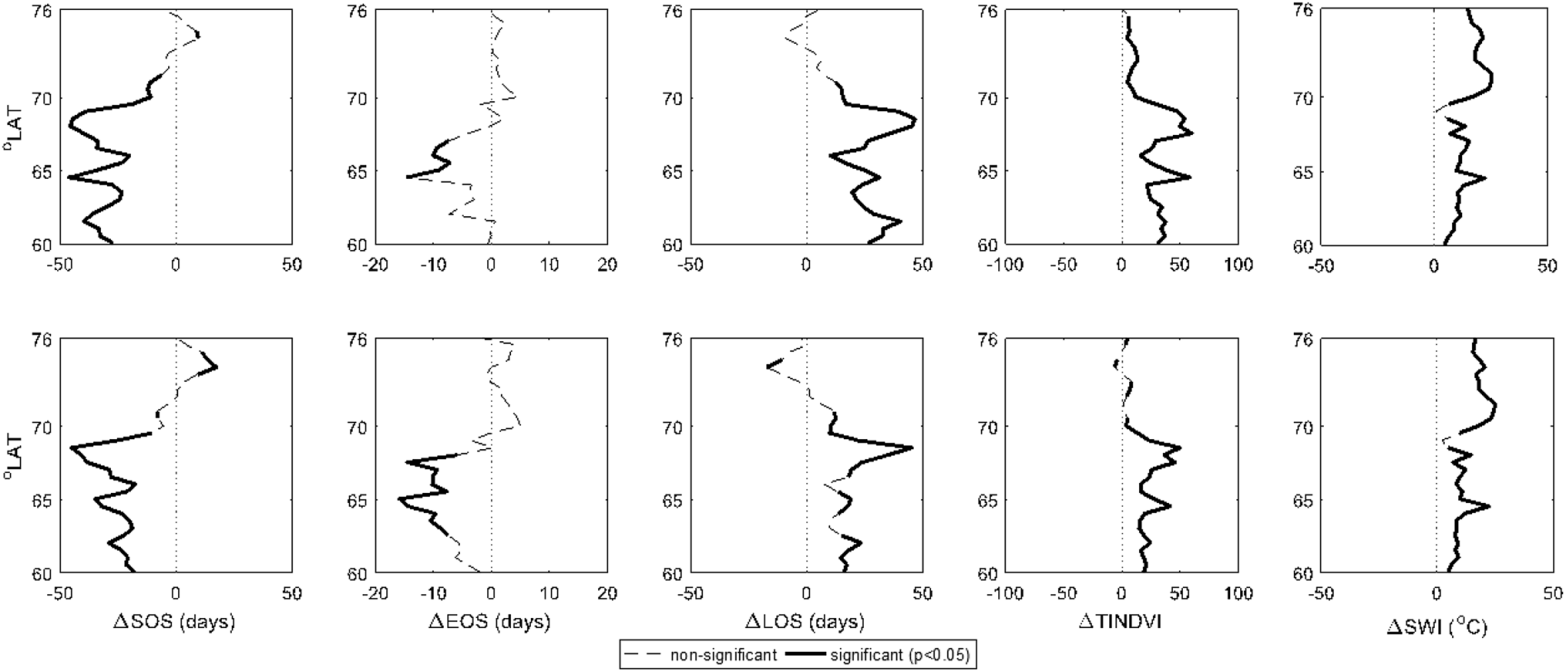
Discrepancies (Δ) between vegetated areas of the West and East Greenland, in terms of the dates of the start of growing season (ΔSOS) and end of growing season (ΔEOS), length of growing season (ΔLOS), time-integrated NDVI (ΔTINDVI), and summer warmth index (ΔSWI). The *top row* illustrates results based on the regionally averaged dataset, whereas the *bottom row* is regionally averaged with standardized topography

The results of the ANOVA test on phenology metrics and SWI on the two coasts are presented in Fig. 4. The plots show the difference between West and East Greenland for each parameter of interest along the latitudinal gradient (e.g., SOS_west_ − SOS_east_). Areas where the difference was significant (*p* < 0.05) are marked. Negative values for ΔSOS and ΔEOS indicate earlier SOS and earlier EOS in West Greenland. Positive ΔLOS, ΔTINDVI, and ΔSWI indicate longer growing season, higher time-integrated NDVI, and higher SWI in West Greenland.

Most of the areas in West Greenland have experienced earlier SOS. However, the differences in terms of SOS start to become unpredictable north of 70°N, mostly fluctuating around 0. This is likely due to the weakened effect of the relatively warm West Greenland Current, which guarantees earlier SOS in affected areas in the southwest compared with the analogous areas on the southeast coast. The only area in East Greenland with consistently earlier SOS date was around 74°N.

Differences in EOS between east and west were mostly insignificant. This can be indicative of the determining effect of the fading light conditions on EOS in comparison with temperatures. TINDVI and SWI were both on significantly higher levels in all regions on the west coast.

The possible effects caused by the differing topography of the two coasts can be examined through comparing the ordinary averaging and standardized topography in Fig. 4. As can be seen, the discrepancies between West and East Greenland are not substantially influenced by topographic differences. Such effects are only noticeable around 74°N where the otherwise significantly earlier SOS, longer growing season, and higher TINDVI values in areas higher than 100 m a.s.l. in East Greenland are overshadowed by the effect from the higher proportion of the pixels located in low-lying areas.

### Vertical gradients along the two coasts

**Fig. 5:**
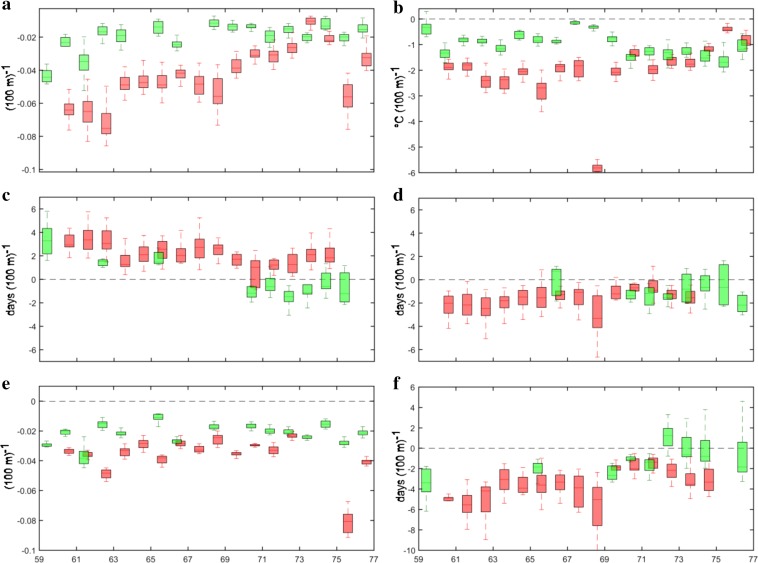
Elevational changes of time-integrated NDVI (**a**), summer warmth index (**b**), timing of the start of season (**c**), timing of the end of season (**d**), maximum annual NDVI (**e**), and length of growing season (**f**) per 100 m along West (*red*) and East (*green*) coasts of Greenland

Figure 5 presents the vertical change rates of phenology metrics and SWI along the latitudinal gradients on the west and east coasts of Greenland. The box plots represent the variations in linear slope values for individual years during the 15-year period. Results are shown only for the locations where the altitudinal changes were significant (*p* < 0.01) over more than 7 years. The results indicate that for SOS and EOS, the effects of elevation were somewhat weaker on the east coast compared to the west, as the effects of elevation become significant in East Greenland only north of 71°N. In West Greenland, the effect of altitude on SOS and EOS had a tendency to diminish from south to the north. On average, SOS date in West Greenland was postponed by 2.3 days (*σ* = 1.2 days) in response to 100 m change in elevation, whereas EOS was advanced by 1.7 days per 100 m (*σ* = 1.2 days). With regard to the maximum annual NDVI, the change rates mostly fluctuate between −0.04 and −0.02 per 100 m across entire Greenland, without any noticeable latitudinal pattern.

### Sensitivity of phenology to sea ice

**Fig. 6:**
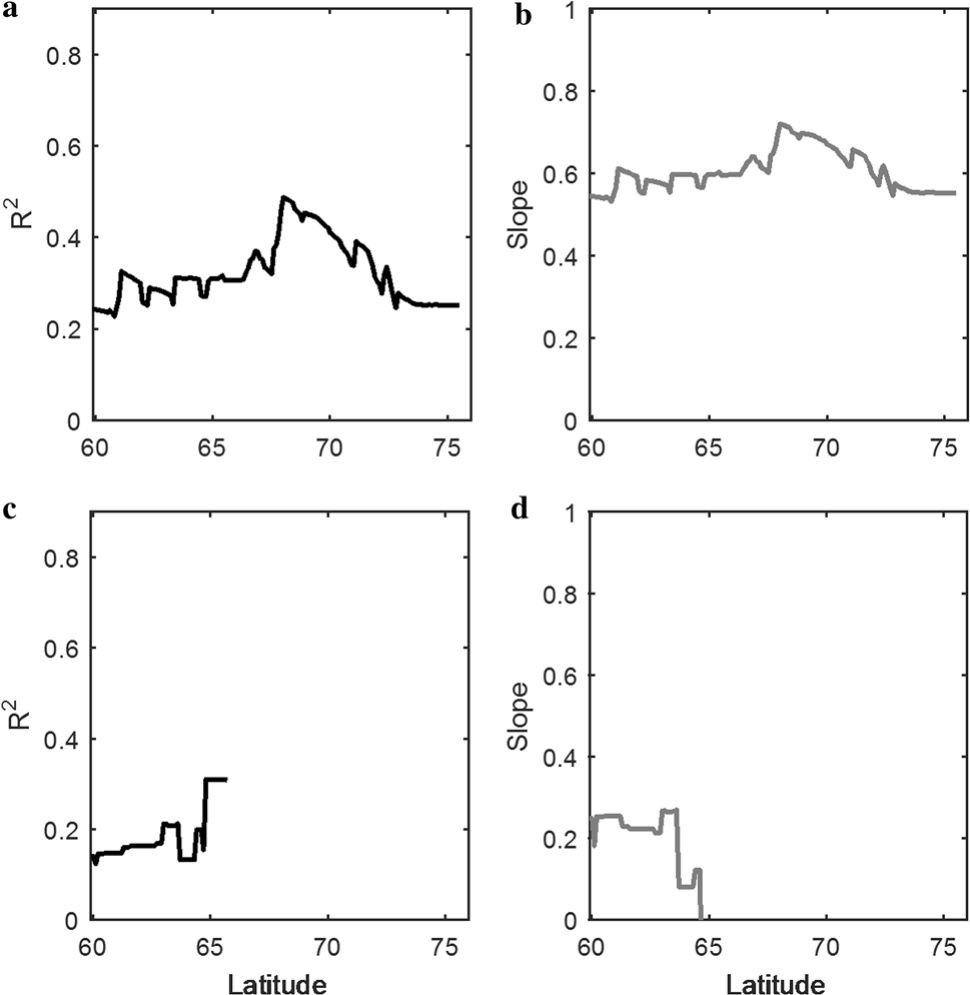
**a**, **b** Sensitivity of the start of season date to sea ice concentrations in West Greenland, as illustrated by average coefficient of determination (**a**) and average linear slope (**b**). **c**, **d** Sensitivity of the start of season date to sea ice concentrations in East Greenland

The sensitivity of regionally averaged SOS to average sea ice concentration during May, estimated based on linear regression, is illustrated in Fig. 6. Average *R* ^2^ and average linear slopes based on the regressions are provided, only taking into account the relationships that were found to be significant (*p* < 0.05). Along the west coast, maximum sensitivity is seen around 68°N, which is reflected in high *R* ^2^ and slope values. On the east coast, however, no significant correlation between regionally averaged SOS and sea ice concentrations was found north of 66°N. The slope values for the west coast were generally higher than for the east, indicating that SOS is more responsive to sea ice dynamics on the west coast of Greenland.

## Discussion

The anomalies of temperatures in Greenland have shown to be linked to the atmospheric circulation patterns. Hanna et al. (2013) have shown that summer temperature anomalies in Greenland are more significantly correlated with blocking patterns over Greenland than with North Atlantic Oscillation Index. The reduction of sea ice has been found to be the cause of these anomalous high-pressure patterns over Greenland during summer (Markus et al. 2009; Liu et al. 2016). Two major observations can be made with regard to our results on the effects of sea ice on phenology in Greenland. The first is the higher sensitivity of the timing of SOS to sea ice concentrations on the west coast of Greenland compared to the east. This can be explained by the fact that due to the incursion of relatively warm currents from the south, the springtime melting of sea ice in Baffin Bay starts from southwest and close to the Greenlandic coast. Along the east coast, however, the southbound East Greenland Current transports large quantities of polar ice throughout the spring, and sea ice concentrations in coastal waters drop only in July to August, depending on the latitude. In other words, year-to-year fluctuations of the spring sea ice concentrations take place close to the Greenlandic coast in the west, whereas these areas are far from the coastline in the east. The second major observation is the increased sensitivity on the west coast between 68 and 70°N. These are the areas where the temporal standard deviation of the sea ice concentrations is generally high near the coastline, whereas further north (south), sea ice concentrations in coastal waters are consistently high (low) during springtime.

Sea ice retreat is shown to contribute to warming in Greenland mainly through two mechanisms: (i) intensified heat transfer from the ocean, mostly to south Greenland, and (ii) increased water vapor content and cloudiness due to more evaporation from the ocean which can increase the downwelling longwave radiation (Markus et al. 2009; Liu et al. 2016). However, caution should be taken when interpreting the mesoscale correlations between sea ice concentrations and SOS as causation. For instance, the presence/absence of sea ice can substantially alter the atmospheric boundary layer conditions during spring by changing the turbulent fluxes of heat and momentum with an impact radius of 200 km (Tetzlaff et al. 2013), which hints at a mesoscale causal effect. Nonetheless, reduced sea ice concentration can itself be a consequence of enhanced downwelling longwave radiation and higher air temperatures at synoptic scales (Belchansky et al. 2004; Maksimovich and Vihma 2012) which can directly impact the melt onset on the land surface independent of the sea ice.

Based on the results, the effects of SWI on time-integrated NDVI and LOS were slightly different in the West compared to East Greenland. It appears that the same levels of SWI are linked to longer growing seasons in East Greenland, and this effect is not linked to topographic differences (Fig. 3d, f). This can be explained by the fact that summers are warmer in West Greenland (Fig. 2). As a result, the same levels of SWI would indicate longer thermal growing seasons in East Greenland. On the other hand, due to higher precipitation, vegetative activity is higher in West Greenland as a whole. Thus, better agreement can be seen between SWI–TINDVI relationships along the two coasts (Fig. 3c, e). The regional averaging approach can also provide insight into the latitudinal dependency of SWI–TINDVI relationship. In both East and West Greenland, the highest TINDVI responses are not observed where the thermal growing season is the longest, but in the southern parts below 61°N. In these areas, although SWI is lower due to cloudiness and higher moisture, the latitudinal control on EOS and higher precipitation allow for longer growing seasons.

## Conclusion

In this study, we used vegetation phenology and a bias-corrected dataset of surface temperatures, all derived from remotely sensed data from MODIS, to investigate the spatial variability of these parameters along environmental gradients in Greenland. The results indicate that all parameters vary significantly from south to north along the latitudinal gradients. Not surprisingly, there is a significant relationship between surface temperatures and vegetation phenology. However, the sensitivity of the vegetation phenology to the surface temperatures may vary from region to region. Moreover, there are significant discrepancies in terms of phenology between analogous regions located on East and West coasts of Greenland, which are presumably linked to the differences in sea ice regimes. With regard to the variations over the altitudinal gradients, we found out that surface temperatures and vegetation phenology do not vary with constant rates in response to elevational changes all across Greenland. Furthermore, in the case of time-integrated NDVI and SWI, significantly different elevational gradients were found on East and West coasts of Greenland, which are probably due to differences in topography as well as the average distance of the ice-free areas to the sea.
